# Geometrical Approaches for the Accurate Identification of Normal Vertical Positions of Sella and Nasion Points in Cephalograms

**DOI:** 10.1155/2022/2705416

**Published:** 2022-11-23

**Authors:** Ashok Kumar Jena, Nida Nayyer, Jitendra Sharan, Binod Kumar Behera, Anand Marya

**Affiliations:** ^1^Department of Dentistry, All India Institute of Medical Sciences, Sijua, Bhubaneswar, Odisha, India; ^2^Department of Community Medicine and Family Medicine, All India Institute of Medical Sciences, Sijua, Bhubaneswar, Odisha, India; ^3^Center for Transdisciplinary Research, Saveetha Dental College, Saveetha Institute of Medical and Technical Science, Saveetha University, Chennai 600077, India; ^4^Department of Orthodontics, Faculty of Dental Medicine, Universitas Airlangga, Surabaya 60115, Indonesia

## Abstract

**Objective:**

The aim of the study is to identify the normal vertical positions of sella (S) and nasion (N) points in subjects with a normal inclination of anterior cranial bases.

**Materials and Methods:**

Lateral cephalograms of 117 subjects who had a normal ∠SN-FH plane (7° ± 1°), ∠SN-palatal plane (9° ± 2°), ∠FH-palatal plane (1° ± 1°), and cranial base angles (131° ± 4°) were included in the study. Various linear and angular parameters and ratios were evaluated to determine the normal vertical positions of S and N points. An unpaired *t*-test was used to identify any significant differences between males and females. The *P* value of 0.05 was considered as the level of significance.

**Results:**

Among subjects with the normal inclinations of SN, FH, and palatal planes and cranial base angle, the mean values of ∠Ar-S-Ptm, ∠S-Ptm-Ar, and ∠S-Ar-Ptm were 59.38° ± 3.52°, 59.70° ± 3.21°, and 60.84° ± 3.56°, respectively, forming an almost equilateral triangle between S, Ar, and Ptm points. The mean values of ∠Ba-S-PNS, ∠S-PNS-Ba, and ∠S-Ba-PNS were 59.56° ± 3.17°, 59.72° ± 3.47°, and 60.76° ± 3.11°, respectively, forming another approximate equilateral triangle between S, Ba, and PNS points. The mean S-FH to N-FH ratio was 0.67 ± 0.06% for the whole sample, but it was significantly greater in males (0.69 ± 0.07%) compared to females (0.65 ± 0.06%) (*P*=0.002).

**Conclusions:**

Two approximate equilateral triangles were formed between S, Ar, and Ptm points; and S, Ba, and PNS points in subjects with normal inclinations of SN, FH, and palatal planes and cranial base angle. The S-FH to N-FH ratio was an excellent guide to locating the normal vertical position of S and N points.

## 1. Introduction

Identification of the accurate sagittal positions of the maxilla and mandible is an important aspect of correct diagnosis and treatment planning in orthodontics. Many cephalometric analyses like ANB angle [1], Wit's appraisal [2], W angle [3], Beta angle [4], Yen angle [5], anteroposterior dysplasia [6], AB plane angle [7], angle of convexity [7], Steiner analysis [8], AXD angle [9], anteroposterior Dysplasia Indicator [10], JYD angle [11], quadrilateral analysis [12], McNamara maxilla-mandibular differential [13], AF-BF distance [14], and APP-BPP distance [15] have been mentioned in the literature for the precise determination of maxillary and mandibular sagittal relationships. Since the introduction of SNA (angle between sella-nasion-point A) and SNB (angle between sella-nasion-point B) angles by Reidel [1], these angles have been the most commonly used and widely accepted cephalometric parameters for the determination of sagittal jaw relationships [16–18]. This is because the anterior cranial base (Sella-Nasion plane, SN plane) is considered to be a very stable reference plane [19, 20], and the identification of landmarks like Sella (S), Nasion (N), Point-A (A), and Point-B (B) is relatively easy and simple. However, the values of SNA, SNB, and ANB angles are affected to a great extent by the length and inclination of the SN plane, making these measurements less reliable in selected cases [21]. Thus, various cephalometric analyses such as Wit's appraisal [2], W angle [3], Beta angle [4], Yen angle [5], anteroposterior dysplasia [6], AXD angle [9], anteroposterior dysplasia indicator [10], JYD angle [11], AF-BF distance [14], and APP-BPP distance [15] have been introduced to overcome these shortcomings. Further, many researchers like Freeman [16], Taylor [17], Mills [22], Camcı, et al. [23], Sadat-Khonsari et al. [24], Johnson [25], and Moore [26] have made an attempt to address the inaccuracies in the measurement of the original SNA, SNB, and ANB angles.

It is a well-established fact that the inclination of the SN plane can significantly affect the severity assessment of the malocclusion [21–23, 27]. The inclination of the SN plane is influenced by the changes in the vertical position of either S or N point or both. Although one study [23] attempted to identify the normal vertical positions of S and N points, it did not provide any systematic method to determine the correct vertical locations of these points. Thus, a well-defined method is needed to identify the true vertical position of S and N points accurately so that correct SNA, SNB, and ANB values can be obtained. The present study describes various geometrical methods to identify the normal vertical positions of S and N points among subjects with normal inclinations of anterior cranial bases.

## 2. Materials and Methods

The study was approved by the Institute Ethics Committee (IEC No. T/IM-NF/Dentistry/21/76) and was designed as per the STROBE statement guidelines. Orthodontic record files of 537 subjects who had either completed or were undergoing comprehensive orthodontic treatment between January, 2015, and July, 2021, were reviewed. Of 537 subjects, 117 (*M* = 54, *F* = 63) fulfilled the selection criteria. The inclusion criteria included the following:

- Good quality pretreatment lateral cephalograms with adequate hard tissue details
- Age between 18–30 years
- Normal inclination of the SN plane to the Frankfort horizontal plane, (7° ± 1°) [28]
- Normal inclination of the SN plane to the palatal plane, (9° ± 2°) [29]
- Normal angle between the Frankfort horizontal plane and the palatal plane, (1° ± 1°) [30]
- Normal cranial base angle (basion-sella-nasion angle, 131° ± 4°) [31]

Subjects with a history of trauma to the maxillofacial region, comprehensive orthodontic treatment, functional jaw orthopedics, surgery for maxilla and mandible, congenital deformities like cleft lip and palate, facial asymmetry, and any systemic disease affecting craniofacial growth were excluded.

**Figure 1:**
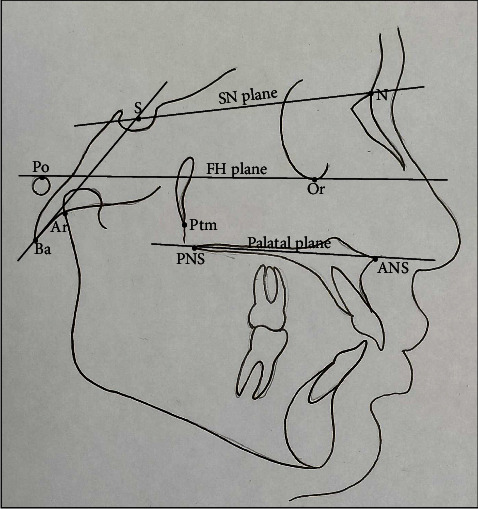
Cephalometric landmarks and reference planes. Landmarks: porion (Po), sella (S), nasion (N), basion (Ba), articulare (Ar), orbitale (Or), pterygomaxillary fissure (Ptm), anterior nasal spine (ANS), and posterior nasal spine (PNS). Reference planes: SN plane, the line joining S and N; FH plane, the line joining Po and Or; palatal plane, the line joining ANS and PNS; S-Ba plane, the line joining S and Ba.

**Figure 2:**
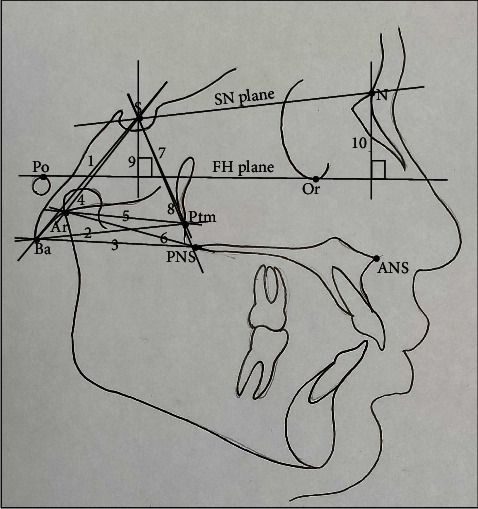
Linear parameters. (1) S-Ba, the linear distance between S and Ba; (2) Ba-Ptm, the linear distance between Ba and Ptm; (3) Ba-PNS, the linear distance between Ba and PNS; (4) S-Ar, the linear distance between S and Ar; (5) Ar-Ptm, the linear distance between Ar and Ptm; (6) Ar-PNS, the linear distance between Ar and PNS; (7) S-Ptm, the linear distance between S and Ptm; (8) S-PNS, the linear distance between S and PNS; (9) S-FH, the linear perpendicular distance from S to FH plane, (10) N-FH, the linear perpendicular distance from N to FH plane. S-FH: N-FH, the ratio of S-FH and N-FH distances in %.

**Figure 3:**
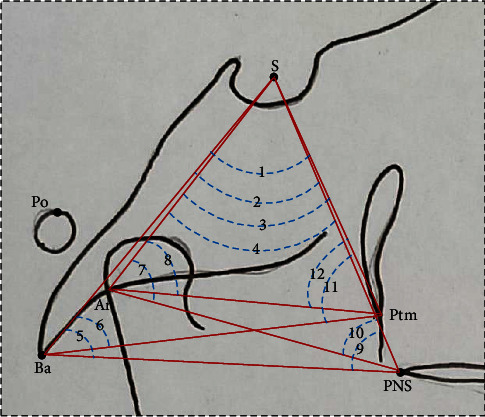
Angular parameters. (1) ∠Ba-S-Ptm, the angle between Ba, S and Ptm; (2) ∠Ba-S-PNS, the angle between Ba, S and PNS; (3) ∠Ar-S-Ptm, the angle between Ar, S and Ptm; (4) ∠Ar-S-PNS, the angle between Ar, S and PNS; (5) ∠S-Ba-PNS, the angle between S, Ba, and PNS; (6) ∠S-Ba-Ptm, the angle between S, Ba, and Ptm; (7) ∠S-Ar-PNS, the angle between S, Ar and PNS; (8) ∠S-Ar-Ptm, the angle between S, Ar and Ptm; (9) ∠S-PNS-Ba, the angle between S, PNS and Ba; (10) ∠S-PNS-Ar, the angle between S, PNS and Ar; (11) ∠S-Ptm-Ba, the angle between S, Ptm and Ba; (12) ∠S-Ptm-Ar, the angle between S, Ptm and Ar.

All cephalograms were recorded with the same machine (NewTom GiANO, Italy) with similar exposure parameters (80Kvp, 10 mAs, and 1.6 seconds). All subjects were positioned with the Frankfort horizontal plane (FH plane) parallel to the floor and teeth in centric occlusion while recording the lateral cephalograms. The heads of all the subjects were kept erect by voice commands. Nine hard tissue landmarks were identified on each cephalogram (Figure 1). The FH plane was considered a reference plane. Ten linear and 12 angular parameters and one ratio were used to establish the normal vertical positions of S and N points (Figures 2 and 3).

All the lateral cephalograms were traced manually by the same evaluator. The linear magnification was corrected and calibrated according to the magnification factor, using the radio-opaque ruler (calibration marker). A digital caliper measured the linear parameters to the nearest 0.01 mm and the angular measurements were done using a protractor to the nearest 0.5°. The assessment of intraobserver errors and the reproducibility of landmark location and measurement errors were analyzed by retracing the 15 randomly selected cephalograms after 3 weeks. The intraobserver reliability of the measurements was calculated by the intraclass correlation coefficient (ICC) for the measurements obtained by the evaluator at both times.

### 2.1. Statistical Analyses

All the statistical analyses were performed in the SPSS software (for Windows 7, version 20, SPSS, Chicago, Ill). Descriptive statistics were used. The Shapiro–Wilk test was used to examine the normality of the data. An unpaired *t*-test determined the significant differences between males and females. The *P* value of 0.05 was considered as the level of significance.

## 3. Results

The ICC for linear and angular measurements ranged from 0.89 to 0.95 and 0.93 to 0.97, respectively, showing excellent reliability between the measurements. The descriptive parameters are mentioned in Table 1. The mean age of the subjects was 22.21 ± 3.92 years. The mean angle between the FH and palatal plane (∠FH-PP), FH and SN plane (∠FH-SN), and palatal and SN plane (∠PP-SN) were 1.06° ± 0.84°, 7.18° ± 0.79°, and 8.26° ± 0.99° respectively. The mean cranial base angle (∠N-S-Ba) was 129.82° ± 2.35°.

Table 2 depicts the values of various linear parameters of all the subjects. All the linear parameters except N-FH distance were significantly greater among males compared to females. S-Ar, Ar-Ptm, and S-Ptm were of almost equal lengths while S-Ba, Ba-PNS, and S-PNS distances were nearly equal to each other. The mean S-Ar, Ar-Ptm, and S-Ptm values were 33.79 ± 3.18 mm, 33.92 ± 2.88 mm, and 34.52 ± 3.04 mm, respectively, thus forming almost an approximate equilateral triangle between the S, Ar, and Ptm points. Similarly, the mean values of S-Ba, Ba-PNS, and S-PNS were 44.10 ± 3.36 mm, 43.75 ± 5.07 mm, and 44.77 ± 3.38 mm, respectively, forming another approximate equilateral triangle between the S, Ba, and PNS points. The mean S-FH to N-FH ratio was 0.67 ± 0.06% for the whole sample, but it was significantly more in males 0.69 ± 0.07%, compared to females 0.65 ± 0.06% (*P* = 0.002).

The details of various angular parameters have been mentioned in Table 3. All the angular parameters were compared between males and females. The mean values of ∠Ar-S-Ptm, ∠S-Ptm-Ar, and ∠S-Ar-Ptm were 59.38° ± 3.52°, 59.70° ± 3.21°, and 60.84° ± 3.56°, respectively, thus forming almost an equilateral triangle between “S, Ar, and Ptm points.” The mean values of ∠Ba-S-PNS, ∠S-PNS-Ba, and ∠S-Ba-PNS were 59.56° ± 3.17°, 59.72° ± 3.47°, and 60.76° ± 3.11°, respectively, forming another approximate equilateral triangle between the S, Ba, and PNS points.

From the results of all linear and angular parameters, it was found that two equilateral triangles can be drawn across the normal craniofacial structure. The first approximate equilateral triangle was formed among the S, Ar, and Ptm points (named as the KUKU triangle) and another between the S, Ba, and PNS points (named as the PUCHU triangle). For both the triangles, the S point was the common vertex point, and we considered the “KUKU triangle” more stable and reliable compared to the “PUCHU triangle.” Hence, the “KUKU triangle” and S-FH to N-FH ratio (S-FH: N-FH) could be used as a reference triangle and ratio, respectively, for the identification of normal vertical positions of S and N points in subjects with aberrant anterior cranial bases.

## 4. Discussion

Effective orthodontic treatment always depends on the accurate diagnosis of malocclusion. Lateral cephalograms are one of the essential aids for the diagnosis of malocclusion. Identifying hard tissue landmarks like Sella (S), Nasion (N), Point-A, and Point-B is very easy. The anterior cranial base completes 90% of its growth in the first 5 years of life [32] and undergoes very minimal change during the growth period [27]; thus, the Sella-Nasion plane (SN plane) is used as a standard reference plane. For this reason, SNA, SNB, and ANB angles are the most accepted and widely used cephalometric parameters for assessing sagittal maxillary and mandibular relationships [16–18]. However, gradual shifting of S and N points takes place throughout the development of craniofacial structures [19], and also, the migration of N point continues for several years parallel to the craniofacial development [33]. To avoid the influence of growth on the inclination of the SN plane, we included the lateral cephalograms of subjects who had completed their craniofacial growths for the present study.

We observed in the present study that when the SN plane is normally inclined to FH and palatal planes, the cranial base angle is within normal limits; two approximately equilateral triangles are formed between S, Ar, and Ptm points (KUKU triangle) and S, Ba, and PNS points (PUCHU triangle) having S point as a common vertex. An important source of influence on the morphology of structures surrounding sella is the growth process of the posterior cranial base [34]. The deflection of the posterior cranial base (S-Ba) could result in a greater alteration in the position of the Ba point compared to the Ar point [34]. However, a compensatory mechanism exists to make up for the position of the glenoid fossa associated with cranial base flexure [34]. The posterior leg (S-Ba) of the cranial base angle (N-S-Ba) can be tipped anteriorly or posteriorly, and according to Andria et al. [35], these are compensated by variable lengths of the posterior cranial base, such as an acute posterior leg that places the mandible forward. This action is negated by a long posterior leg (S-Ba) that places both the mandible (Ar) and basion (Ba) posteriorly and vice-versa. Thus, we considered the KUKU triangle more stable and reliable than the PUCHU triangle for the identification of the normal vertical position of sella point. We also observed that the distance from the S point to the FH plane is approximately 69% and 65% of the distance from the N point to the FH plane among male and female subjects, respectively. Similar to our observation, Camcı and Salmanpour also reported a 69.62% S-FH to N-FH ratio among subjects with normal SN-FH angles [23].

A significant variation in the angle between SN and FH planes can mislead orthodontists in interpreting abnormal jaw positions, even if the jaws are normally positioned. Thus, one must be cognizant of the SN-FH angulation if the SN plane is used as a reference plane for cephalometric analyses. There has been no study so far in the literature assessing the normal relationships between points S, Ar, Ba, Ptm, and PNS. This is the first attempt at exploring these measurements of the craniofacial skeleton. The KUKU triangle and S-FH to N-FH ratio can be used as standard reference triangle and ratio to determine the normal vertical position of Sella and Nasion points among subjects with abnormal inclinations of anterior cranial bases.

### 4.1. Limitations and Future Recommendations

Although there are newer alternatives to the conventional methods of cephalometric analysis such as the use of reduced FOV CT, this study was an audit of existing records [36–39]. Hence, future studies can be planned to explore comparisons between newer methods and our proposed methods to establish reliability.

## 5. Conclusions

The following conclusions were drawn from the present study:

1. The triangles formed between S, Ar, and Ptm points and S, Ba, and PNS points were almost equilateral among subjects with normal inclinations of SN, FH, and palatal planes.
2. An almost equilateral triangle between S, Ar and Ptm points indicated the normal vertical position of the S point.
3. The vertical distance of the S point was nearly 67% of the vertical distance of the N point from the FH plane.

## Figures and Tables

**Table 1:** Various descriptive parameters of the subjects.

Variables	Gender	Values mean ± SD	Significance (*P* value) Males vs females
Age (yrs)	Males	22.06 ± 3.86	0.704^NS^
	Females	22.33 ± 3.99	
	Total	22.21 ± 3.92	

∠FH-PP (°)	Males	1.17 ± 0.88	0.186^NS^
	Females	0.97 ± 0.80	
	Total	1.06 ± 0.84	

∠FH-SN (°)	Males	7.02 ± 0.84	0.033^∗^
	Females	7.33 ± 0.73	
	Total	7.18 ± 0.79	

∠PP-SN (°)	Males	8.23 ± 1.08	0.585^NS^
	Females	8.33 ± 0.92	
	Total	8.26 ± 0.99	

Cranial base angle (∠N-S-Ba) (°)	Males	129.74 ± 2.26	0.736^NS^
	Females	129.89 ± 2.45	
	Total	129.82 ± 2.35	

N, nasion; S, sella; Ba, basion; FH, Frankfort horizontal plane; PP, palatal plane; SN, SN plane. (Yr) = year, ∠ = angle, (°) = degree, SD = standard deviation, NS = nonsignificant, ^∗^ =  *P* < 0.05.

**Table 2:** Various linear parameters and ratio used to determine the normal vertical positions of S and N points.

Variables	Gender	Values mean ± SD	Significance (*P* value) Males vs females
S-Ba distance (mm)	Males	45.69 ± 3.13	0.000^∗∗∗^
	Females	42.75 ± 2.96	
	Total	44.10 ± 3.36	

S-Ar distance (mm)	Males	35.46 ± 2.89	0.000^∗∗∗^
	Females	32.37 ± 2.70	
	Total	33.79 ± 3.18	

S-Ptm distance (mm)	Males	35.98 ± 3.04	0.000^∗∗∗^
	Females	33.27 ± 2.44	
	Total	34.52 ± 3.04	

S-PNS distance (mm)	Males	46.44 ± 3.38	0.000^∗∗∗^
	Females	43.33 ± 2.68	
	Total	44.77 ± 3.38	

Ar-Ptm distance (mm)	Males	35.26 ± 3.01	0.000^∗∗∗^
	Females	32.78 ± 2.21	
	Total	33.92 ± 2.88	

Ar-PNS distance (mm)	Males	38.78 ± 3.23	0.000^∗∗∗^
	Females	36.22 ± 2.55	
	Total	37.40 ± 3.14	

Ba-Ptm distance (mm)	Males	43.80 ± 3.39	0.000^∗∗∗^
	Females	41.54 ± 2.81	
	Total	42.58 ± 3.25	

Ba-PNS distance (mm)	Males	44.70 ± 6.66	0.060^∗^
	Females	42.94 ± 2.96	
	Total	43.75 ± 5.07	

S-FH distance (mm)	Males	18.28 ± 2.26	0.000^∗∗∗^
	Females	16.70 ± 1.93	
	Total	17.43 ± 2.23	

N-FH distance (mm)	Males	26.33 ± 2.41	0.053^NS^
	Females	25.51 ± 2.16	
	Total	25.89 ± 2.30	

S-FH:N-FH ratio (%)	Males	0.69 ± 0.07	0.002^∗∗^
	Females	0.65 ± 0.06	
	Total	0.67 ± 0.06	

Ba, basion; Ar, articulare; S, sella; Ptm, pterygomaxillary fissure; PNS, posterior nasal spine; FH, Frankfort horizontal plane. SD = standard deviation, NS = nonsignificant, ^∗^ =  *P* < 0.05,^∗∗^ =  *P* < 0.01, ^∗∗∗^ = *P* < 0.001.

**Table 3:** Various angular parameters used for the determination of normal vertical positions of S point.

Variables	Gender	Values Mean ± SD	Significance (*P* value) Males vs females
∠Ba-S-Ptm (°)	Males	63.28 ± 4.52	0.088^NS^
	Females	64.69 ± 4.28	
	Total	64.04 ± 4.43	

∠Ba-S-PNS (°)	Males	59.43 ± 2.98	0.677^NS^
	Females	59.68 ± 3.35	
	Total	59.56 ± 3.17	

∠Ar-S-Ptm (°)	Males	58.78 ± 3.53	0.089^NS^
	Females	59.89 ± 3.46	
	Total	59.38 ± 3.52	

∠Ar-S-PNS (°)	Males	54.00 ± 3.18	0.291^NS^
	Females	54.67 ± 3.54	
	Total	54.36 ± 3.38	

∠S-Ptm-Ba (°)	Males	69.63 ± 5.36	0.437^NS^
	Females	68.96 ± 3.77	
	Total	69.27 ± 4.56	

∠S-Ptm-Ar (°)	Males	60.04 ± 3.29	0.284^NS^
	Females	59.40 ± 3.13	
	Total	59.70 ± 3.21	

∠S-PNS-Ba (°)	Males	59.80 ± 2.87	0.811^NS^
	Females	59.65 ± 3.93	
	Total	59.72 ± 3.47	

∠S-PNS-Ar (°)	Males	48.51 ± 3.73	0.095^NS^
	Females	47.26 ± 4.21	
	Total	47.83 ± 4.03	

∠S-Ba-Ptm (°)	Males	47.08 ± 4.03	0.262^NS^
	Females	46.34 ± 3.08	
	Total	46.86 ± 3.55	

∠S-Ar-Ptm (°)	Males	61.11 ± 3.63	0.460^NS^
	Females	60.61 ± 3.52	
	Total	60.84 ± 3.56	

∠S-Ba-PNS (°)	Males	60.90 ± 2.81	0.659^NS^
	Females	60.65 ± 3.36	
	Total	60.76 ± 3.11	

∠S-Ar-PNS (°)	Males	77.55 ± 4.36	0.559^NS^
	Females	78.07 ± 5.03	
	Total	77.83 ± 4.72	

Ba, basion; Ar, articulare; S, sella; Ptm, Pterygomaxillary fissure; PNS, posterior nasal spine. ∠ = angle, (°) = degree, SD = standard deviation, NS = nonsignificant.

## Data Availability

All data related to the study can be provided on reasonable request.
